# 

**Published:** 1861-04

**Authors:** 


### RECEIPTS NOT HERETOFORE ACKNOWLEDGED.

T. B. Dever, Ill., $2; J. H. Leal, Ill., R. Q. Wilson, Ind., P. M. McFarland, Ill., Rowley Morris, Wis., each $2 for vol. 4; John Allen, Ill., vol. 3, $2; L. P. Cheny, Ill., vol. 3, $2; K. E. Rich, Ill., vol. 4, $2; J. S. D. Comstock, Ind., vol. 4, $2; F. R. Millerd, Wis., vol. 4, $2; A. D. Coe, Ind., vol. 3, $2; Hosea Davis, Ill., half vols. 3 and 4, $3; D. Prince, Ill., vol. 3, $2; J. W. Thayer, Ill., vol. 4, $2; Jas. G. Conly, Wis., vol. 4, $2; Wm. Cargen Wis., vol. 4, $2.

